# The effect of diet and radiation on the bacterial symbiome of the melon fly, *Zeugodacus cucurbitae* (Coquillett)

**DOI:** 10.1186/s12896-019-0578-7

**Published:** 2019-12-18

**Authors:** Elias D. Asimakis, Mahfuza Khan, Panagiota Stathopoulou, Carlos Caceres, Kostas Bourtzis, George Tsiamis

**Affiliations:** 1 Department of Environmental Engineering, University of Patras, 2 Seferi St., 30131 Agrinio, Greece; 2 Insect Biotechnology Division, Institute of Food and Radiation Biology (IFRB), Atomic Energy Research Establishment (AERE), Ganak bari, Savar, Dhaka 1349 Bangladesh; 3 Insect Pest Control Laboratory, Joint FAO/IAEA Division of Nuclear Techniques in Food and Agriculture, International Centre, P.O. Box 100, 1400 Vienna, Austria

**Keywords:** SIT, Melon fly, *16S rRNA* gene, Next generation sequencing, NGS

## Abstract

**Background:**

Symbiotic bacteria contribute to a multitude of important biological functions such as nutrition and reproduction and affect multiple physiological factors like fitness and longevity in their insect hosts. The melon fly, *Zeugodacus cucurbitae* (Coquillett), is an important agricultural pest that affects a variety of cultivated plants belonging mostly to the Cucurbitaceae family. It is considered invasive and widespread in many parts of the world. Several approaches are currently being considered for the management of its populations including the environmentally friendly and effective sterile insect technique (SIT), as a component of an integrated pest management (IPM) strategy. In the present study, we examined the effect of diet and radiation on the bacterial symbiome of *Z*. *cucurbitae* flies with the use of Next Generation Sequencing technologies.

**Results:**

Melon flies were reared on two diets at the larval stage, an artificial bran-based diet and on sweet gourd, which affected significantly the development of the bacterial profiles. Significant differentiation was also observed based on gender. The effect of radiation was mostly diet dependent, with irradiated melon flies reared on the bran diet exhibiting a significant reduction in species diversity and richness compared to their non-irradiated controls. Changes in the bacterial symbiome of the irradiated melon flies included a drastic reduction in the number of sequences affiliated with members of *Citrobacter*, *Raoultella*, and Enterobacteriaceae. At the same time, an increase was observed for members of *Enterobacter*, *Providencia* and *Morganella*. Interestingly, the irradiated male melon flies reared on sweet gourd showed a clear differentiation compared to their non-irradiated controls, namely a significant reduction in species richness and minor differences in the relative abundance for members of *Enterobacter* and *Providencia*.

**Conclusions:**

The two diets in conjunction with the irradiation affected significantly the formation of the bacterial symbiome. Melon flies reared on the bran-based artificial diet displayed significant changes in the bacterial symbiome upon irradiation, in all aspects, including species richness, diversity and composition. When reared on sweet gourd, significant changes occurred to male samples due to radiation, only in terms of species richness.

## Background

Insects harbour a variety of microbes, which are associated mostly with their reproductive and digestive tissues and range from obligate symbionts, which are crucial for the normal function of the host, to facultative symbionts, whose presence is not essential for the host [1, 2]. Depending on the type of interaction, symbionts can be further distinguished into commensals or parasites, which have either neutral or negative impact on hosts, or mutualistic which contribute to important aspects of host biology [1]. Symbiotic bacteria, especially those of the reproductive and digestive tissue, influence a variety of important physiological properties of their insect hosts. Most importantly, they provide essential nutrients, including amino acids, vitamins, carbon and nitrogen compounds, promoting development and improving host fitness, which ultimately could result in increased resistance to parasites, pathogens, pesticides and heat stress. Other aspects of host physiology include speciation, through the coevolution with their hosts, communication and reproduction, by causing embryo mortality by means of cytoplasmic incompatibility (CI), shift in the sex ratio of progeny in favour of females through feminization, parthenogenesis and male killing or increased fecundity [1, 3–19].

The genus *Zeugodacus* (Hendel) contains approximately 192 species [20] that are mostly distributed in regions of Asia and Oceania with a few of them occurring in eastern China and Japan. *Zeugodacus* (*Bactrocera*) *cucurbitae* (Coquillett) is considered an invasive species due to its introduction in many regions of Africa (East and West), in islands of the Indian Ocean and Hawaii [20–24]. The melon fly is considered an important agricultural pest affecting a variety of cultivated fruit and vegetable plants. It is mainly polyphagous, but oligophagous populations have been found in Thailand, Malaysia and France (Reunion Island, Indian Ocean) [25–28]. Its hosts were initially estimated at 81 species [23] but their number was later reduced to 45 well-recorded species that belong to 9 different families, most of them members of the Cucurbitaceae family [20].

The sterile insect technique (SIT) is an important component of an IPM strategy and is based on the release of sterile male individuals that mate with females from wild populations thus reducing the chances of producing offspring, ultimately leading to the suppression or the eradication of the target local population [29, 30]. In order to control insect pests using SIT, the production of large numbers of high quality sterile adult males is needed [29]. However, mass rearing, irradiation, handling and transport may affect the quality of the mass produced sterile insects, and this may be associated with their impact on symbiotic bacterial communities [4, 31, 32]. At the same time, insect gut bacteria can be exploited as a means of enhancing fitness and mating competitiveness of mass reared male insects [33–38].

It is therefore important to expand our knowledge of the impact of diet and radiation on the symbiotic bacterial communities of insect pest species targeted with SIT, in order to enhance its application. To that end, we used Next Generation Sequencing (NGS) technologies to examine the structure of the bacterial symbiome of irradiated and non-irradiated adult *Z*. *cucurbitae* flies that were reared on two distinct diets, one artificial and one based on a natural host.

## Results

Bacterial community composition and diversity of *Z. cucurbitae* laboratory populations, kept on an artificial larval diet based on wheat bran and on a natural host (sweet gourd) and treated with irradiation at a 50 Gy dose, were investigated by *16S rRNA* gene amplicon sequencing. In total 220,955 reads after quality filtering were used for analysis (Table 1), providing high coverage (97–98%) of the existing bacterial diversity based on the Good’s coverage index (Table 2).

**Table 1:** Summary of the samples that were analyzed. The samples include irradiated male and female individuals that were raised on bran or sweet gourd and their respective non-irradiated controls

No.	ID	Gender	Age	Fertility	Diet	No. of reads
1	F_IR_BR	Female	15 days old	Irradiated (50 Gy)	Bran	27,391
2	M_IR_BR	Male	15 days old	Irradiated (50 Gy)	Bran	26,519
3	F_NIR_BR	Female	15 days old	Non-irradiated	Bran	27,751
4	M_NIR_BR	Male	15 days old	Non-irradiated	Bran	25,589
5	F_IR_SG	Female	15 days old	Irradiated (50 Gy)	Sweet gourd	29,512
6	M_IR_SG	Male	15 days old	Irradiated (50 Gy)	Sweet gourd	25,085
7	F_NIR_SG	Female	15 days old	Non-irradiated	Sweet gourd	32,638
8	M_NIR_SG	Male	15 days old	Non-irradiated	Sweet gourd	26,470
					**Total**	220,955

**Table 2:** Species richness and diversity were estimated with the use of four indices, two in each case. The high values of the Good’s coverage index show good representation of the existing diversity in each sample. For each index, the value of the standard error is also shown

			Species richness indices		Species diversity indices	
No.	ID	Good’s coverage	Chao1	Ace	Shannon	Simpson reciprocal
1	F_IR_BR	0.98	406.27 ± 9.74^a^	453.35 ± 7.74^a^	2.95 ± 0.00^a^	3.60 ± 0.01^a^
2	M_IR_BR	0.98	333.04 ± 4.63^b^	366.96 ± 5.54^b^	3.31 ± 0.01^b^	5.71 ± 0.02^b^
3	F_NIR_BR	0.97	583.58 ± 12.45^c^	594.74 ± 8.81^c^	4.55 ± 0.00^c^	12.38 ± 0.02^c^
4	M_NIR_BR	0.98	471.42 ± 7.25^d^	488.04 ± 6.94^a^	4.41 ± 0.00^d^	11.07 ± 0.02^d^
5	F_IR_SG	0.97	622.23 ± 13.95^c^	747.99 ± 13.92^d^	4.55 ± 0.01^c^	10.62 ± 0.02^e^
6	M_IR_SG	0.97	522.74 ± 8.63^e^	555.49 ± 5.04^e^	4.90 ± 0.00^e^	11.57 ± 0.04^f^
7	F_NIR_SG	0.98	448.09 ± 13.97^d^	483.93 ± 11.6^a^	4.00 ± 0.00^f^	6.91 ± 0.02^g^
8	M_NIR_SG	0.97	611.78 ± 9.95^c^	666.66 ± 11.69^f^	4.90 ± 0.00^e^	12.01 ± 0.04^h^

For each diversity index, ANOVAs followed by the Tukey HSD test, (*P* value < 0.05 ). Significant differences are indicated by different letters

### Diet and / or irradiation dependent changes

**Fig. 1:**
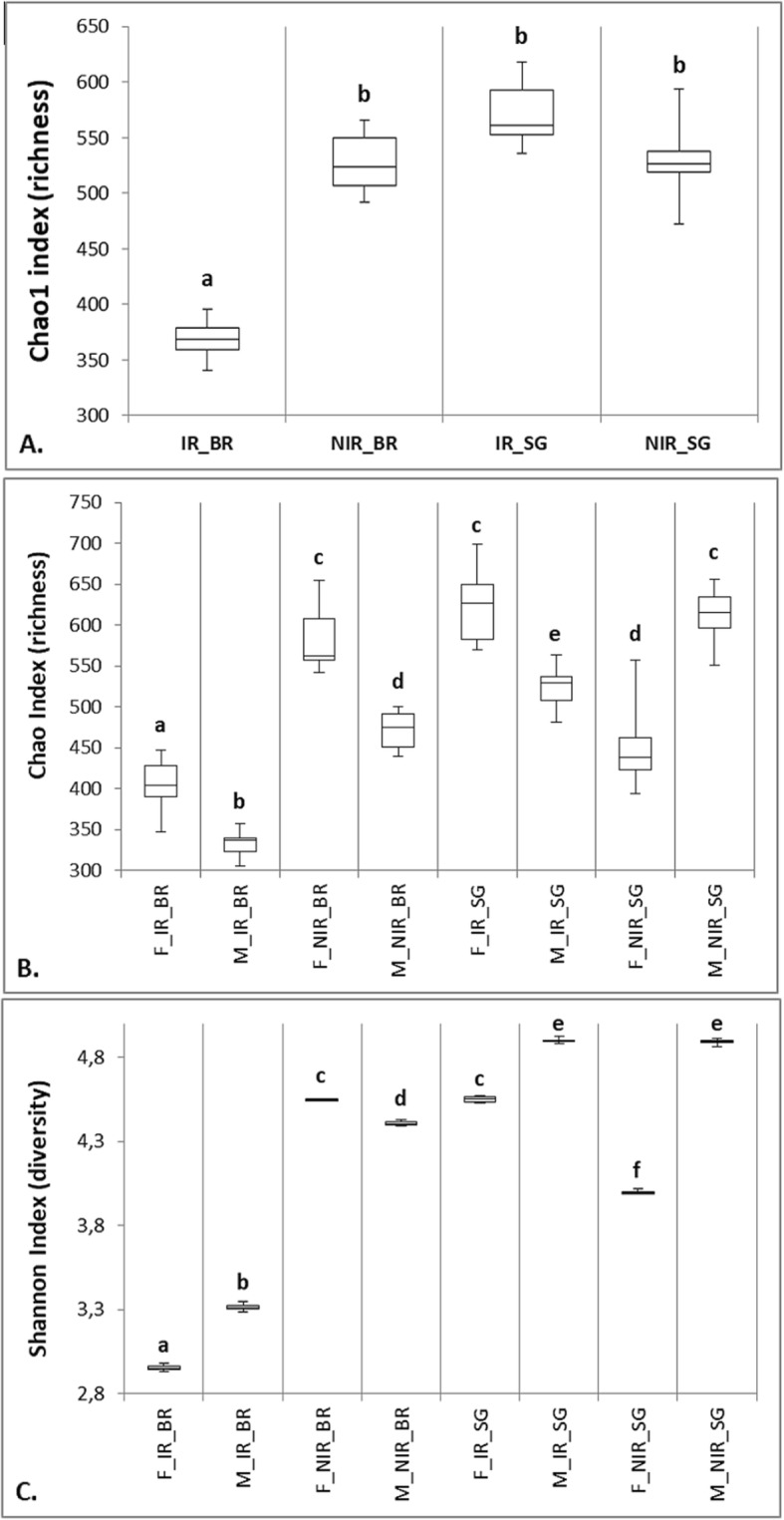
Species richness and diversity indices based on the Chao1 and Shannon indices. **a** Species richness of *Z. cucurbitae* samples reared on bran-diet, irradiated (IR_BR) and non-irradiated (NIR_BR), and samples reared on sweet gourd, irradiated (IR_SG) and non-irradiated (NIR_SG). **b** Species richness of female (F) and male (M) samples reared on bran-diet (BR) and sweet gourd (SG) before (NIR) and after irradiation (IR). **c** Diversity index of female (F) and male (M) samples reared on bran-diet (BR) and sweet gourd (SG) before (NIR) and after irradiation (IR). Boxes denote the interquartile range, the line within the box is the median, and whiskers extend to the most extreme values

Diet and / or irradiation-dependent changes in bacterial diversity and composition were observed. Non-irradiated samples reared on the artificial bran-based diet (NIR_BR) exhibited similar species diversity and richness compared to non-irradiated controls reared on sweet gourd (NIR_SG) (t-test; df: 18; *p* < 1; Fig. 1). Interestingly, the non-irradiated samples kept on the bran-based diet (NIR_BR), exhibited statistically higher diversity and richness than the irradiated samples (IR_BR) (t-test; df:18; *p* < 0.027; Fig. 1). The non-irradiated flies that were reared on sweet gourd (NIR_SG) displayed similar richness compared to their irradiated counterparts (IR_SG) (t-test; *p* < 0.3; Fig. 1). Also, the irradiated samples reared on bran (IR_BR) were characterized by both lower richness and diversity compared to irradiated samples reared on sweet gourd (IR_SG) (t-test; df:18; *p* < 0.027; Fig. 1).

**Fig. 2:**
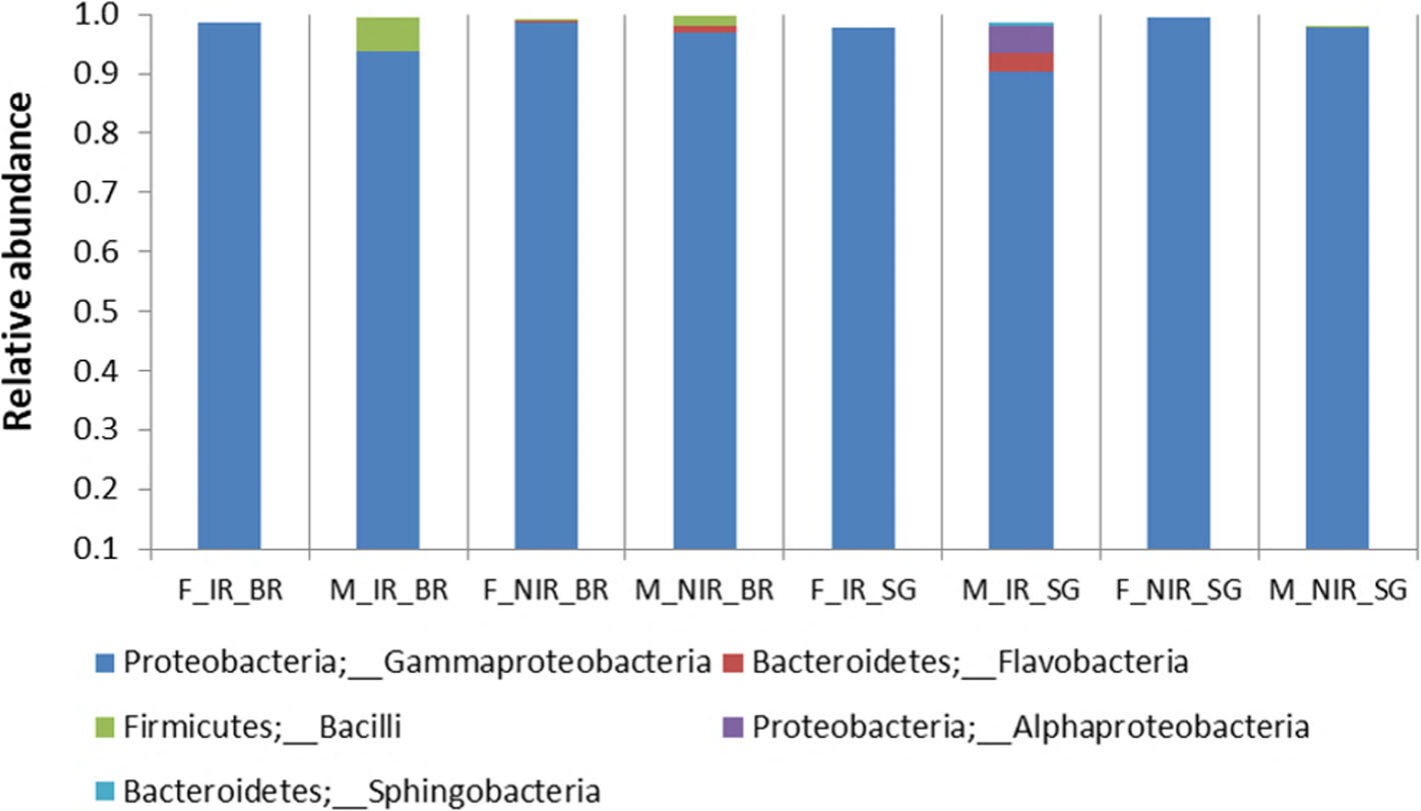
Relative abundance of the most dominant phyla of all samples examined

**Fig. 3:**
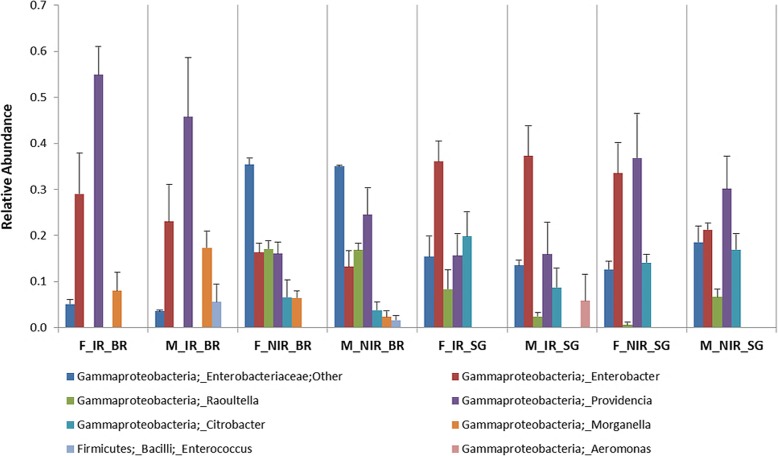
Relative abundance of the most dominant taxa among all samples examined

The bacterial OTU composition appeared to be relatively uniform in all samples at higher taxonomic levels. The most dominant phylum was Proteobacteria exhibiting high relative abundance (94–100%), with Gammaproteobacteria being the most prevalent class (90–100%) followed by Alphaproteobacteria (4%). In some samples, Firmicutes (2–6%) and Bacteroidetes (1–4%) were also detected but to a lesser degree, with Firmicutes represented by only one class, Bacilli (2–6%), and Bacteroidetes by two, Flavobacteria (1–3%) and Sphingobacteria (1%; Fig. 2). The non-irradiated controls of the two diets displayed differences in bacterial composition. Non-irradiated samples reared on wheat bran (NIR_BR) showed higher relative abundance in sequences assigned to *Raoultella* and other Enterobacteriaceae and lower in *Enterobacter*, *Providencia* and *Citrobacter* sequences than samples reared on sweet gourd (NIR_SG), which were also characterized by the absence of *Morganella* (Fig. 3). The irradiated melon flies reared on wheat bran (IR_BR) exhibited significant changes in the bacterial composition from their respective non-irradiated controls (NIR_BR). These changes include a substantial increase in the relative abundance of *Providencia*, *Enterobacter* and *Morganella* assigned sequences (Kruskal-Wallis; *p* < 0.001; *p* < 0.02; *p* < 0.03), and a significant decrease in the relative abundance of members of the Enterobacteriaceae family (Kruskal-Wallis; *p* < 0.001) and a complete absence of sequences affiliated to *Citrobacter* or *Raoultella* (Fig. 3). On the other hand, the irradiated melon flies reared on sweet gourd (IR_SG) did not exhibit significant differences in bacterial composition from their non-irradiated controls (NIR_SG) (Fig. 3), except for a decline in the relative abundance of sequences assigned to *Providencia* (Fig. 3).

**Fig. 4:**
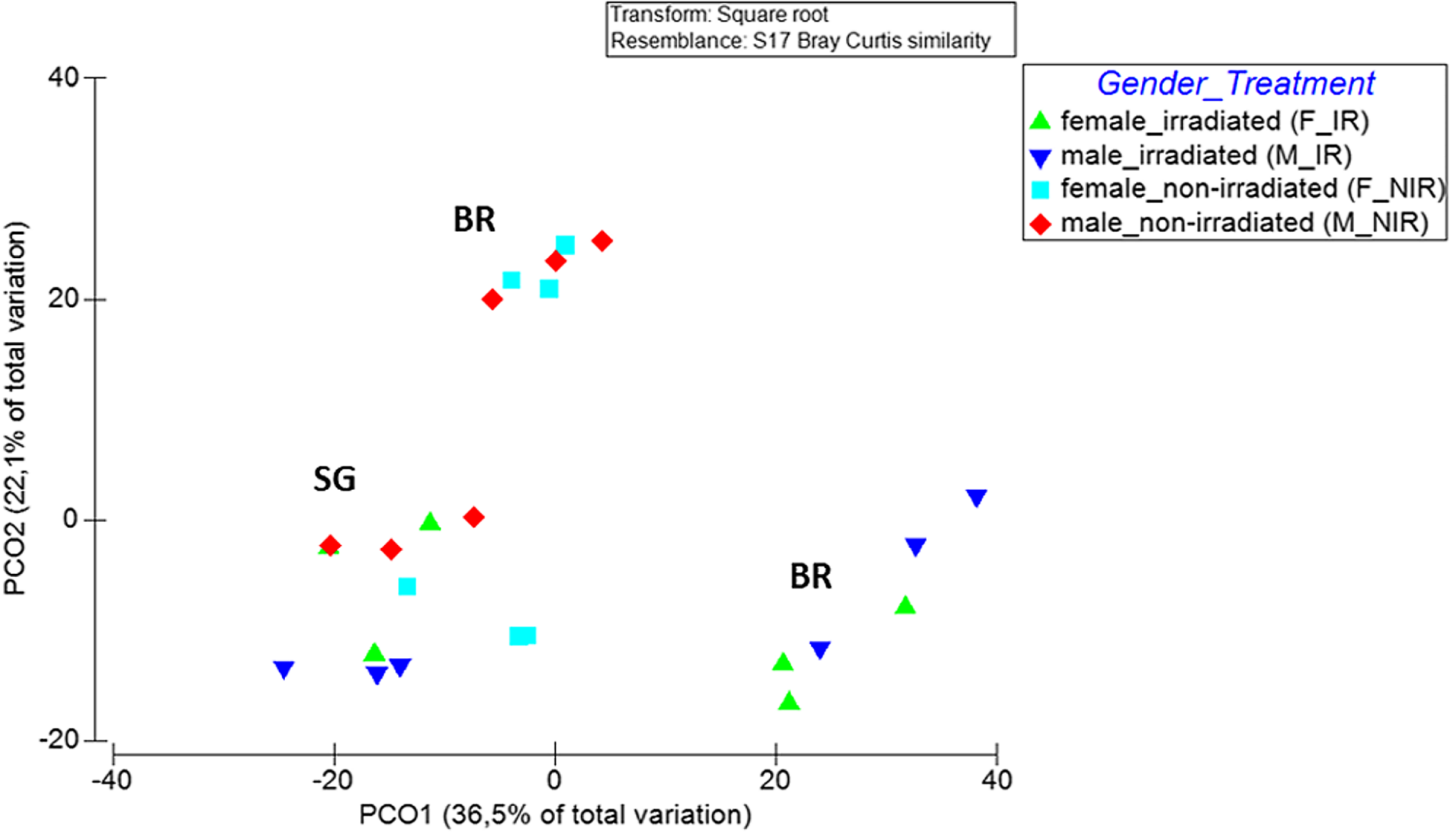
Principal Coordinates Analysis (PCoA) of bacterial communities based on relative abundances of OTUs with originations from males, females, irradiated, non-irradiated, bran-based and sweet gourd samples. (BR = bran; SG = sweet gourd)

**Fig. 5:**
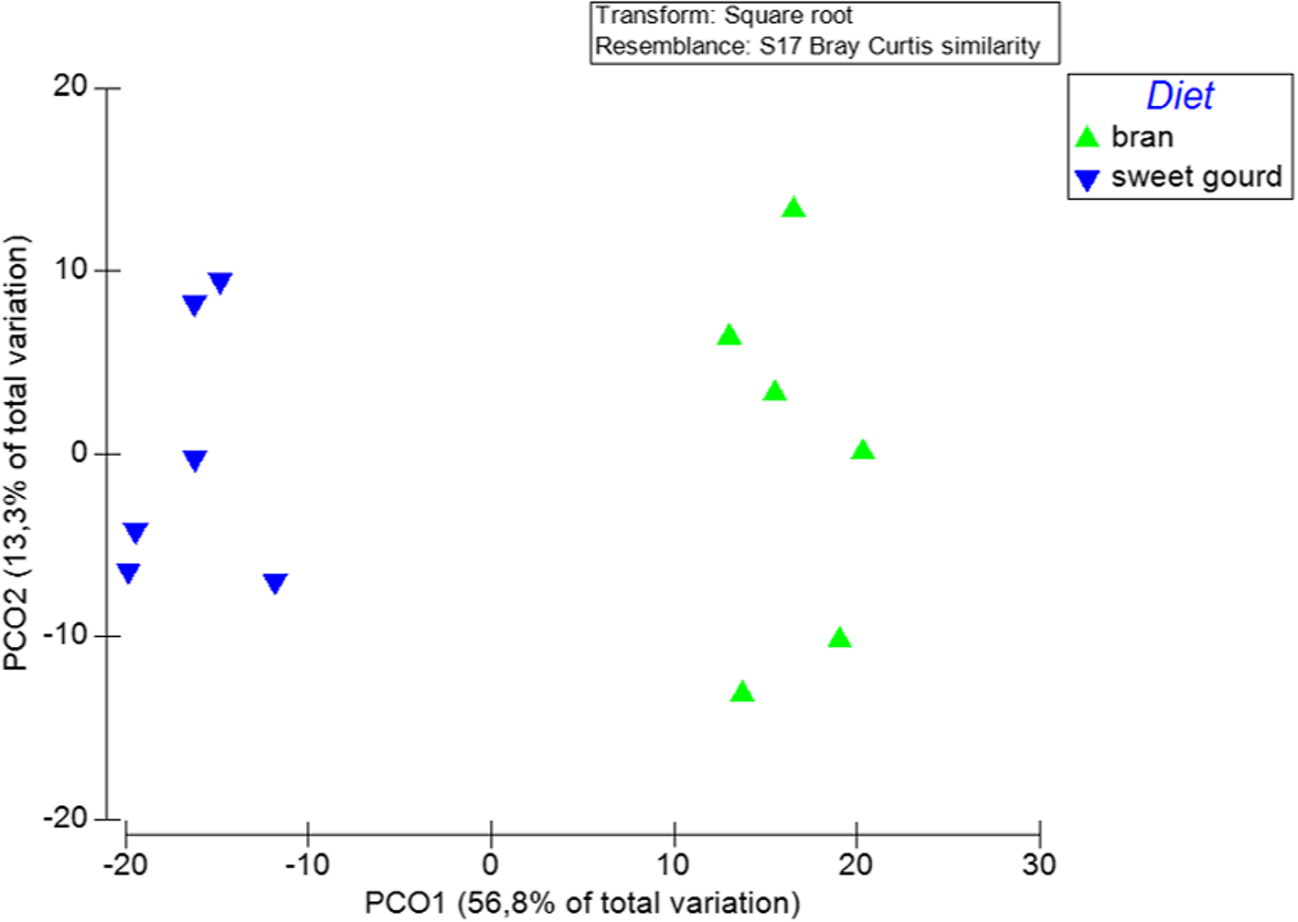
Principal Coordinates Analysis (PCoA) of bacterial communities based on relative abundances of OTUs with originations from non-irradiated samples (PERMANOVA; *p* < 0.002)

With respect to beta-diversity, bacterial communities were strongly clustered according to the larval diet and the irradiation treatment (PERMANOVA; *p* < 0.001; Table 3). PCoA revealed the formation of distinct clusters between the two larval diets used, wheat bran and sweet gourd (PERMANOVA; *p* < 0.001; Figs. 4 and 5). Furthermore, bacterial communities of samples raised on artificial wheat bran were strongly clustered according to their irradiation status, with the PCoA plot explaining 58.6% of the existing variance (Fig. 4). On the other hand, *Z. cucurbitae* flies that were reared on sweet gourd grouped together regardless of their irradiation status (PERMANOVA; *p* < 0.1; Fig. 4).

**Table 3:** PERMANOVA table of results for all three factors and their combinations. Statistically significant differences (*p*<0.05) can be seen in bold letters in all three factors separately and in the combination of treatment and diet

Source	df	SS	MS	Pseudo-F	P(perm)	Unique perms	(MC)
Treatment	1	3109.7	3109.7	8.174	**0.001**	999	0.001
Diet	1	5904.4	5904.4	15.52	**0.001**	998	0.001
Gender	1	998.91	998.91	2.6257	**0.03**	999	0.031
Treatment x Diet	1	3598.6	3598.6	9.4593	**0.001**	997	0.001
Treatment x Gender	1	481.13	481.13	1.2647	0.236	998	0.273
Diet x Gender	1	613.49	613.49	1.6126	0.149	997	0.161
Treat x Diet x Gender	1	596.73	596.73	1.5686	0.169	998	0.174
Res	16	6087	380.43				
Total	23	21,390

### Gender dependent changes in diversity and composition

Gender dependent differences in species diversity and composition were observed between samples. In general, female exhibited higher richness, except for the non-irradiated on sweet gourd (NIR_SG) (Fig. 1b), whereas diversity was larger in males than females, except for the non-irradiated on the bran diet (NIR_BR) (Fig. 1c). In more detail, the non-irradiated females reared on the bran diet (F_NIR_BR) exhibited higher species richness and diversity indices than the males (M_NIR_BR) (t-test; *p* < 0.001; Fig. 1b and c). On the other hand, samples reared on sweet gourd exhibited the opposite pattern with the non-irradiated males (M_NIR_SG), showing higher species richness and diversity indices than the females (F_NIR_SG) (t-test; *p* < 0.001; Fig. 1b and c). Interestingly, the bran-based male and female irradiated samples exhibited lower species richness and diversity indices than the non-irradiated males and females (t-test; df: 18; *p* < 0.027; Fig. 1b and c). For the samples reared on sweet gourd, the irradiated males (M_IR_SG) exhibit lower species richness (t-test;df: 18; *p* < 0.027) but equal diversity indices (t-test; df: 18; *p* < 0.9) when compared to the non-irradiated (M_NIR_SG) (Fig. 1b and c). Interestingly, the female irradiated samples kept on sweet gourd (F_IR_SG) exhibited a higher species richness and diversity when compared to the non-irradiated (F_NIR_SG) (t-test; df: 18; *p* < 0.027; Fig. 1b and c).

The bran-based non-irradiated female samples (F_NIR_BR) show a higher relative abundance of sequences affiliated to *Citrobacter* and *Morganella* and a lower relative abundance with sequences affiliated to *Providencia* when compared with the bran-based non-irradiated male samples (M_NIR_BR). For the irradiated samples we observed the presence of sequences affiliated with *Enterococcus* in the male samples (M_IR_BR), coupled with an increase in the relative abundance of *Morganella* sequences (Fig. 3). Interestingly, in male and female irradiated samples reared on bran, sequences affiliated to *Citrobacter* and *Raoutella* were not detected while members of *Enterobacter* and *Providencia* tended to increase, but with sequences assigned to Enterobacteriaceae decreasing when compared to male and female non-irradiated samples (Fig. 3). For the sweet gourd, we observed a decrease in the sequences affiliated with *Raoutella* in the female non-irradiated samples (F_NIR_SG) when compared with the male samples (M_NIR_SG). Interestingly, in the female irradiated samples a decrease in the relative abundance of *Providencia* affiliated sequences was observed coupled with an increase in the members of *Raoutella* when compared to the female non-irradiated samples (F_NIR_SG) (Fig. 3). For the male irradiated samples, a decrease was observed with sequences assigned to *Providencia*, *Citrobacter*, *Raoutella*, while an increase was observed in the relative abundance of *Enterobacter* and *Aeromonas* (Fig. 3).

**Fig. 6:**
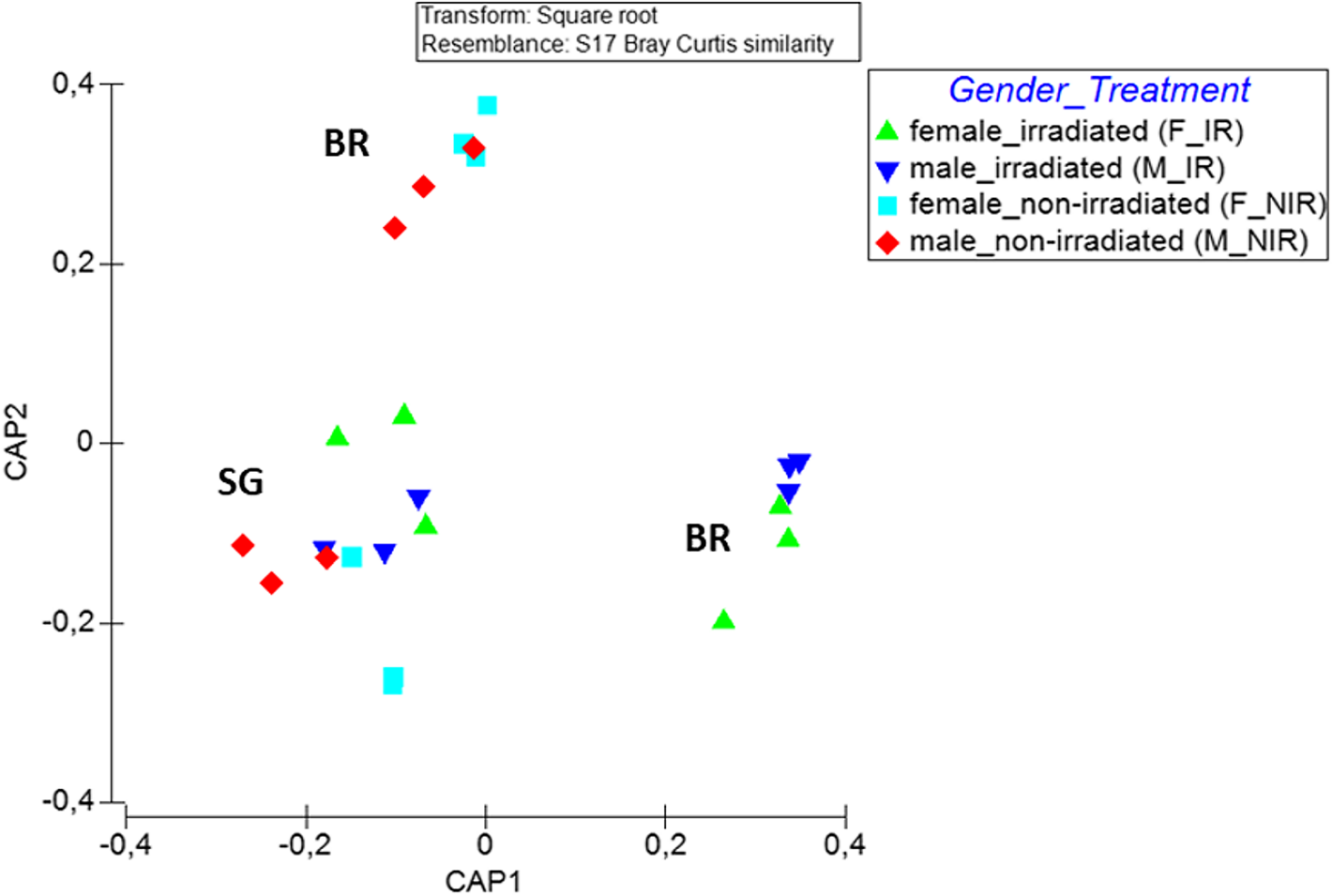
Canonical analysis of principal coordinates (CAP) ordinations of bacterial communities based on relative abundances of bacterial OTUs from *Z. cucurbitae* samples examined, based on different diets (sweet gourd (SG) and wheat bran (BR)), irradiation treatment (non-irradiated (NIR) and irradiated (IR)) and gender (male (M) and female (F)). The constrained ordinations show maximized differences among the two diets and the irradiated against the non-irradiated samples (trace_Q_m’HQ_m_ statistics (2.52837; *p* < 0.001))

As suggested by the aforementioned differences in diversity and composition, bacterial communities seem to be affected by the sex of the melon flies (PERMANOVA; *p* < 0.03; Fig. 6), with non-irradiated female and male controls (NIR_F and NIR_M) forming separate but closely related clusters depending on their diet (Fig. 6). Further clustering was observed when the irradiation treatment was taken into account (PERMANOVA; *p* < 0.03; Fig. 6).

## Discussion

The present study examined the effect of larval diet, gender and irradiation on the symbiotic bacterial communities of *Z*. *cucurbitae* laboratory flies. Our results indicate that the application of irradiation to two distinct larval diets leads to the formation of different bacterial profiles. Different bacterial profiles were also observed between samples belonging to different genders. Furthermore, statistically significant differences were observed after irradiation treatment in the two diets examined and between genders. Irradiation had a detrimental effect on the formation of bacterial communities especially in samples reared on bran whereas irradiated flies reared on sweet gourd showed mixed responses but overall managed to retain higher levels of richness and diversity, with small changes in OTU composition.

Differences in insect microbiota due to the application of distinct diets have been previously described [31, 39–47]. These studies focused on the comparison of symbiotic communities between wild populations and laboratory strains that were reared on various artificial diets or between lab populations reared on different artificial diets. In the case of wild populations of diverse *Drosophila* species, analysis of samples of fruit-feeding flies and samples of flower-feeders revealed the development of significantly different bacterial microbiomes between the two diets [41]. Variability among *D. suzukii* samples of different developmental stages, reared on fruits and artificial diet was also reported [45]. One of the most important functions of mutualistic symbionts is the provision of nutritional components from their diet to their insect-hosts, by hydrolysis, using specialized enzymes [13]. Different bacterial groups are capable of digesting different nutrients. In this way, as the composition of the symbiotic community changes, so does the ability of hosts to exploit diets with different nutritional properties. Such changes in bacterial communities have been previously described in different developmental stages of *Bactrocera* flies, with immature and adult flies developing different microbiota, due to different dietary habits and needs [48–50]. In these cases, larvae that mostly require diets rich in carbohydrates are characterized by different microbial communities from adults that are in need of both sugars and proteins. The development of different microbiotas between the non-irradiated samples that were reared on the artificial wheat bran diet and on sweet gourd could be due to the different bacteria that exist in each diet provided, since the majority of symbionts are usually acquired from the environment [51]. It is also possible that prevalence of certain symbionts is favoured by differences in the nutritional components of the two diets that require different types of bacteria in order to be processed.

Our study indicated that Proteobacteria were the dominant phylum in all samples examined, with Gammaproteobacteria being the most abundant class. Sequences belonging to Firmicutes and Bacteroidetes were also identified but in a lesser degree. A recent cultivation-dependent study of the midgut bacterial composition characterized from wild *Z*. *cucurbitae* flies displayed a similar picture, with Proteobacteria being the most abundant followed by Firmicutes and Actinobacteria, while Enterobacteriaceae were the most prevalent family [52]. The dominant species were from the genera *Enterobacter*, *Klebsiella*, *Citrobacter*, *Bacillus* and *Providencia*. All these genera, except for *Bacillus*, were also identified in our study showing significant relative abundance with the exception of *Klebsiella*, which was found only in irradiated samples reared on sweet gourd, with low relative abundance (1–2%). Interestingly, a second cultivation-dependent approach that used gut tissue from a laboratory population indicated that Firmicutes were the most abundant phylum, with species belonging to *Bacillus* [53]. However, female melon flies were characterized by the presence of *Morganella* species which were also identified in our work.

Similarly to previous studies in *Ceratitis capitata* [31, 32, 54], radiation altered the structure of the microbiota of *Z. cucurbitae*, causing reduction in bacterial symbiont richness and diversity. These alterations appeared to be much stronger in samples reared on the artificial wheat bran diet. In those samples, the relative abundance of genera like *Raoultella*, *Citrobacter* and other Enterobacteriaceae decreased, in contrast to members belonging to *Providencia*, *Morganella* and *Enterobacter*. Interestingly, certain strains belonging to *Providencia* and *Morganella* are known pathogens to flies or even humans [55–57]. Increase in the relative abundance could be associated with their emancipation into the hemolymph of the irradiated flies since treatment with radiation damages their gut tissue [32], although the degree to which pathogens and compounds enter the irradiated gut, and the existence of any gut dysfunction remains to be determined. Further characterization of these strains would be required to fully decipher their exact role in melon flies. Increased presence of potentially pathogenic strains, belonging to the genus *Pseudomonas*, was also reported in mass reared Medfly after irradiation [31]. At the same time, members of Enterobacteriaceae like *Providencia*, *Citrobacter* and *Enterobacter* function as attractants for both male and female *Z. cucurbitae* [52]. Additionally, *Citrobacter, Klebsiella* and *Enterobacter* contain species with probiotic properties to insects and arthropods [4, 7, 31, 33–38, 58]. Usually, these probiotic effects include improved fitness, longevity and increased reproductive capabilities for irradiated individuals resulting in increased competitiveness against wild populations [34, 35]. In *Z. cucurbitae,* enrichment of the larval diet with *Enterobacter* resulted in improved pupal weight, morphological indices, and adult survival rate [38] as well as increased pupal and adult productivity and faster development, particularly of males, in its closely related species *C. capitata* [33].

Differences based on gender in the symbiotic bacterial profiles of the non-irradiated controls varied between the two diets, with females reared on bran showing higher richness and diversity compared to males, and the exact opposite trend when reared on sweet gourd. Differences were also observed in OTU composition in both diets. Previously, the gut tissue of wild *Bactrocera dorsalis* females was described with lower species richness, higher diversity and differences in bacterial composition compared to males [48]. Treatment with irradiation resulted in lower richness and lower or equal diversity in male samples, in both diets, compared to their non-irradiated controls. Reduction in diversity and differences in the composition of the bacterial communities between irradiated and non-irradiated male samples was also described in studies with *C. capitata*, with irradiated gut samples containing the genera *Salmonella*, *Citrobacter*, *Providencia*, *Morganella*, *Enterobacter*, *Klebsiella and Pectobacterium* [31]. The majority of those genera were also identified in irradiated males in our work, as mentioned previously. On the contrary, female melon flies showed mixed trends after radiation treatment, depending on their diet. Irradiated females reared on bran followed the same trend as both irradiated males while those reared on sweet gourd the exact opposite trend, which resulted in an unusual increase in both richness and diversity compared to the non-irradiated sample. Changes in the symbiotic bacterial communities of male melon flies due to radiation could be of greater importance since males are targets for the application of the SIT. Significant alterations can be seen in irradiated male samples reared on both diets, but those on bran are affected in a larger degree. As mentioned earlier, these effects of radiation to the composition of the symbiotic bacterial communities could be possibly reversed by enriching the diet with nutrients or certain bacteria with probiotic properties.

## Conclusions

Diet was found to strongly affect the structure of the microbiota in *Z*. *cucurbitae* flies. Significant differentiation in the microbiota was also observed based on the sex of the flies, but to a lesser extent. The effect of radiation was diet-dependent with sweet gourd exhibiting minor changes in the bacterial profile between irradiated and non-irradiated melon flies. A strong effect was observed on flies that were reared on an artificial bran-based diet and was characterized by a decrease in both bacterial richness and diversity, with bacterial genera like *Raoultella* and *Citrobacter* being highly reduced while sequences affiliated to members of *Providencia*, *Morganella* and *Enterobacter* were increased. Gender dependent radiation effects were mostly observed in terms of species richness and diversity, with males showing considerable losses and females mixed trends.

## Methods

### Rearing conditions and sample preparation

*Zeugodacus cucurbitae* flies used in the present study originated from a population collected from infested sweet gourd (*Cucurbita maxima* Duchesne) and have been reared for more than 500 generations at IBD, IFRB, AERE (Insect Biotechnology Division, Institute of Food and Radiation Biology, Atomic Energy Research Establishment), Ganak bari, Savar, Dhaka, Bangladesh. Larvae were reared on two distinct diets: (a) an artificial larval diet based on wheat bran which was sterilized at 80 °C for 3 days and is usually used in the artificial rearing of *Z. cucurbitae* ((wheat bran (26%), sugar (12%), Brewer’s yeast (3.6%), sodium benzoate (0.4%) and water (58%)) for one generation and (b) a natural host, sweet gourd, in order to examine the effect of a natural and artificial diet on the bacterial symbiome of *Z. cucurbitae*. Pupae were irradiated 24–48 h before adult emergence at 50 Gy, a dose capable of inducing 100% sterility in both males and females [59], with the use of a cobalt-60 gamma radiation source of IFRB. Adult flies were reared on artificial diets containing casein, yeast extract and sugar in a ratio of 1:1:2. Prior to extraction, the insects were surface sterilized.

### DNA extraction, PCR amplification and sample purification

DNA extraction was performed following a simplified CTAB protocol [60]. Extracted samples were diluted in sterile deionized water and stored in − 20 °C. Each processed sample consisted of fifteen (*n* = 15) whole adult flies (15 days old) divided into three replicates of five flies. A fragment of approximately 460 bp belonging to the V3-V4 region of the bacterial *16S rRNA* gene was amplified by PCR using the universal primer set U341F-MiSeq 5′-CCTACG GGR SGC AGC AG-3′ and 805R-MiSeq 5′-GA CTA CHV GGG TAT CTA ATC C-3′ [61]. Amplification was performed using KAPA HiFi HotStart PCR Kit (Kapa Biosystems). Each 25 μl reaction contained 5 μl of KAPA HiFi Fidelity Buffer (5X), 0.7 μl of dNTPs solution (10 mM each), 0.7 μl of each primer solution (10 μM), 0.3 μl of KAPA HiFi HotStart DNA Polymerase solution (1 U/μl), 1 μl from the template DNA solution and was finalized with 16.6 μl of sterile deionized water. The PCR protocol was comprised of an initial denaturation step at 95 °C for 3 min, followed by 30 cycles of denaturation at 98 °C for 20 s, annealing at 60 °C for 15 s and extension at 72 °C for 45 s. The reaction was terminated with a final extension step at 72 °C for 1 min. For each set of PCR reactions performed, the appropriate negative and positive controls were also prepared. From each reaction, 5 μl were loaded on a 1.5% agarose gel and separated by electrophoresis. The approximately 550 bp amplification product (size increase due to the incorporation of the 50-mer Illumina primers) was visualized in Bio-Rad’s Gel Doc™ XR+ system. Positive PCR products were purified with a 20% PEG, 2.5 M NaCl solution, centrifuged at 14.000 x *g* for 20 min and the precipitate was washed twice with 125 μl of a 70% v/v ethanol solution and centrifuged at 14.000 x *g* for 10 min as previously described [62]. The dried precipitates were suspended in 15 μl of sterile deionized water and the concentration was measured with a Quawell Q5000 micro-volume UV-Vis spectrophotometer.

### Indexing PCR and sample purification

The purified PCR products were diluted to a final concentration of 10 ng/μl and submitted to indexing PCR in order to incorporate the Illumina index primers to their sequence. During indexing PCR, each sample was amplified with a unique combination of index primers. Amplification was performed in 50 μl reactions using the KAPA HiFi HotStart PCR Kit. Each reaction contained 10 μl of KAPA HiFi Fidelity Buffer (5X), 1.5 μl of dNTPs solution (10 mM each), 5 μl of the forward index primer (10 μM), 5 μl of the reverse index primer (10 μΜ), 1 μl of KAPA HiFi HotStart DNA Polymerase (1 U/μl), 2 μl from the diluted PCR product (10 ng/μl) and 25.5 μl of sterile deionized water. The PCR program was comprised of an initial denaturation step at 95 °C for 3 min, followed by 8 cycles of denaturation at 95 °C for 30 s, annealing at 55 °C for 30 s and extension at 72 °C for 30 s. The reaction was terminated with a final extension step at 72 °C for 5 min. The resulting amplicons were purified using Macherey-Nagel’s NucleoMag® NGS Clean-up and Size Selection kit according to the manufacturer’s recommendations. Purified samples were suspended in 30 μl of sterile deionized water and their concentration was measured with a Quawell Q5000 micro-volume UV-Vis spectrophotometer. All samples were diluted to a final concentration of 8 nM and mixed equimolarly.

### Illumina sequencing and data analysis

The library was sequenced on an Illumina MiSeq sequencing platform by IMGM SA. The sequencing results for each sample were retrieved in two FASTq files and were assembled with the use of PANDAseq v2.7 assembler [63]. Chimeric contigs were identified and removed with UCHIME [64] which is contained within the software package Qiime v1.9.1 [65]. Taxonomic assignment and alpha-diversity analysis were also performed with Qiime. Taxonomy was assigned using the SILVA *16S rRNA* gene database (release 119) [66]. Species richness was estimated with Chao1 [67] and ACE indices [68] whereas species diversity was calculated with the use of Shannon’s and Simpson’s reciprocal (1/D) indices. Rarefaction analyses were performed starting with 250 sequences/sample as a minimum subsample, 5000 sequences/sample as the maximum, a 250-sequence increment/step and 10 iterations/step. Alpha-diversity comparisons were performed by t-test using Qiime including Holm-Bonferroni sequential correction. Between-sample diversity was calculated using Bray-Curtis similarity [69] on square root transformed data and principal coordinates analysis (PCoA) [70] was performed on the resulting distance matrix. Canonical analysis of principal coordinates (CAP) [71] was based on 999 permutation tests. Statistically significant differences between samples were identified with permutational multivariate analysis of variance (PERMANOVA) [72] using 999 permutations and Monte Carlo tests. Beta-diversity calculations were performed with Primer6+ [73]. OTU comparisons were performed using the non-parametric Kruskal-Wallis Rank Sum test, and the Mann-Whitney test. The obtained significance values were corrected for multiple testing using the Benjamini-Hochberg method [74]. 16S *rRNA* gene sequences reported in this study have been deposited in NCBI under BioProject number PRJNA487513.

## Data Availability

The datasets used and/or analyzed during the current study are available in NCBI.

## Abbreviations

CAP: Canonical Analysis of Principal coordinates
CI: Cytoplasmic Incompatibility
F_IR_BR: Female Irradiated Bran
F_IR_SG: Female Irradiated Sweet Gourd
F_NIR_BR: Female Non-irradiated Bran
F_NIR_SG: Female Non-irradiated Sweet Gourd
IPM: Integrated Pest Management
IR_BR: Irradiated Bran
IR_SG: Irradiated Sweet Gourd
M_IR_BR: Male Irradiated Bran
M_IR_SG: Male Irradiated Sweet Gourd
M_NIR_BR: Male Non-irradiated Bran
M_NIR_SG: Male Non-irradiated Sweet Gourd
NGS: Next Generation Sequencing
NIR_BR: Non-irradiated Bran
NIR_F: Non-irradiated Female
NIR_M: Non-irradiated Male
NIR_SG: Non-irradiated Sweet Gourd
OTU: Operational Taxonomic Unit
PCoA: Principal Coordinates Analysis
SIT: Sterile Insect Technique
